# Draft Genome Sequence of Ligilactobacillus salivarius FFIG58, Isolated from the Intestinal Tract of Wakame-Fed Pig

**DOI:** 10.1128/MRA.00839-20

**Published:** 2020-08-20

**Authors:** Binghui Zhou, Leonardo Albarracin, Yuki Masumizu, Yuhki Indo, M. Aminul Islam, Valeria Garcia-Castillo, Wakako Ikeda-Ohtsubo, Yoshihito Suda, Hisashi Aso, Julio Villena, Haruki Kitazawa

**Affiliations:** a Food and Feed Immunology Group, Graduate School of Agricultural Science, Tohoku University, Sendai, Japan; b Livestock Immunology Unit, International Education and Research Center for Food Agricultural Immunology, Graduate School of Agricultural Science, Tohoku University, Sendai, Japan; c Laboratory of Immunobiotechnology, Reference Centre for Lactobacilli (CERELA-CONICET), Tucuman, Argentina; d Scientific Computing Laboratory, Computer Science Department, Faculty of Exact Sciences and Technology, National University of Tucuman, Tucuman, Argentina; e Department of Medicine, Faculty of Veterinary Science, Bangladesh Agricultural University, Mymensingh, Bangladesh; f Department of Food, Agriculture, and Environment, Miyagi University, Sendai, Japan; Broad Institute

## Abstract

Ligilactobacillus salivarius FFIG58 was isolated from the intestine of a wakame-fed pig and sequenced with an Illumina HiSeq system. FFIG58 genome sequencing revealed a genome size of 1,984,180 bp, with 1,994 protein-coding genes and a GC content of 32.9%. This draft genome sequence will contribute to a better understanding of the porcine gut microbiome.

## ANNOUNCEMENT

Ligilactobacillus salivarius (basonym: Lactobacillus salivarius) (1) is considered an important member of the swine intestinal microbiota (2, 3) because of its capacity to protect against intestinal infections through inhibition of the growth of pathogens, competition for mucosal binding sites, or modulation of the immune system (4–7). Our previous work demonstrated that feeding pigs wakame (*Undaria pinnatifida*), economically important edible algae in Asian countries (8), was able to modify the gastrointestinal microbiota, inducing a significant increase in the abundance of lactobacilli (9). A library of L. salivarius strains isolated from the intestine of pigs fed wakame was constructed (9), and we investigated the capacities of those strains to modulate the innate antiviral immune response in porcine intestinal epithelial (PIE) cells. Among the strains evaluated, L. salivarius FFIG58 had a remarkable capacity to improve interferon beta levels in PIE cells after Toll-like receptor 3 activation and to protect against rotavirus infection (9).

A single colony of FFIG58 cultured on MRS agar (Oxoid, Cambridge, UK) plates was inoculated into MRS broth and incubated at 37°C for 12 h. Genomic DNA isolation was performed by using a lysozyme lysis buffer (75 mM NaCl, 20 mM EDTA, 20 mM Tris-HCl [pH 7.5], 10 mg/ml lysozyme), chloroform-isoamyl alcohol separation, and isopropanol precipitation method, as described previously (10). The genomic DNA of L. salivarius FFIG58 was sequenced with an Illumina HiSeq platform using the 2 × 300-bp paired-end read sequencing protocol. The paired-end sequencing library was prepared using the TruSeq DNA HT sample preparation kit (Illumina) according to the manufacturer’s protocol. The paired-end reads were filtered with prinseq-lite v.0.20.4 (min_qual_mean, 20; min_len, 75) (11). After filtering, the reads were assembled using A5-miseq v.201690825 (12) with default parameters. The sequencing protocol generated 59.7× mean coverage of the genome. The FFIG58 draft genome sequence contains 222 contigs, with an average GC content of 32.9% and a total estimated size of 1,984,180 bp.

Sequencing results were analyzed using various software programs with default settings, unless otherwise specified. Gene prediction and annotation were performed using the NCBI Prokaryotic Genome Annotation Pipeline (PGAP) v.4.12 (13) and the Rapid Annotations using Subsystems Technology (RAST) server (14). This genome contained 2,041 open reading frames, with 1,994 DNA coding sequences, 36 tRNA coding sequences, and 11 rRNA coding sequences.

RAST system category distribution showed 223 subsystems. The FFIG58 genome contains enzymes for carbohydrate metabolism that are not found in the genomes of L. salivarius strains of porcine origin, such as strains JCM1046 (15), CP400 (16), and ZLS006 (17). Among those, the d-galactarate, d-glucarate, and d-glycerate transport and catabolism enzymes could be involved in the ability of FFIG58 to grow efficiently in wakame-based media (9). A gene for a choloylglycine hydrolase was also detected, which could be involved in the ability of L. salivarius to survive in the gastrointestinal tract (2). In addition, genes of the SecA2-SecY2 system, which have been proposed as important factors for adaptation to the intestinal tract in L. salivarius strains isolated from pigs (2), were found in the genome of FFIG58.

The draft genome sequence of L. salivarius FFIG58 will be useful for further studies of specific genetic features and could contribute to a better understanding of the intestinal porcine microbiome.

### Data availability.

This whole-genome shotgun project has been deposited in DDBJ/ENA/GenBank under the accession number JACBJR000000000. The version described in this paper is version JACBJR010000000. The SRA/DRA/ERA accession number is SRR12128965. The BioProject and BioSample numbers are PRJNA643532 and SAMN15418202, respectively.
